# Cognitive Flexibility and Inhibition in Individuals with Age-Related Hearing Loss

**DOI:** 10.3390/geriatrics6010022

**Published:** 2021-03-05

**Authors:** Shraddha A. Shende, Lydia T. Nguyen, Elizabeth A. Lydon, Fatima T. Husain, Raksha A. Mudar

**Affiliations:** 1 Department of Speech and Hearing Science, University of Illinois at Urbana-Champaign, Champaign, IL 61820, USA; sshende2@illinois.edu (S.A.S.); elydon2@illinois.edu (E.A.L.); husainf@illinois.edu (F.T.H.); 2 Neuroscience Program, University of Illinois at Urbana-Champaign, Urbana, IL 61801, USA; lnguyen@in2l.com; 3 Beckman Institute for Advanced Science and Technology, Urbana, IL 61801, USA

**Keywords:** age-related hearing loss, speech-in-noise recognition, hearing, cognitive flexibility, inhibition

## Abstract

Growing evidence suggests alterations in cognitive control processes in individuals with varying degrees of age-related hearing loss (ARHL); however, alterations in those with unaided mild ARHL are understudied. The current study examined two cognitive control processes, cognitive flexibility, and inhibition, in 21 older adults with unaided mild ARHL and 18 age- and education-matched normal hearing (NH) controls. All participants underwent comprehensive audiological and cognitive evaluations including Trail Making Test-B, Verbal Fluency, Stroop, and two Go/NoGo tasks. Group differences in cognitive flexibility and inhibition as well as associations between peripheral and central hearing ability and measures of cognitive flexibility and inhibition were investigated. Findings revealed that the ARHL group took significantly longer to complete the Stroop task and had higher error rates on NoGo trials on both Go/NoGo tasks relative to the NH controls. Additionally, poorer peripheral and central hearing were associated with poorer cognitive flexibility and inhibitory control. Our findings suggest slower and more inefficient inhibitory control in the mild ARHL group relative to the NH group and add to decades of research on the association between hearing and cognition.

## 1. Introduction

Age-related hearing loss (ARHL) is the gradual loss of hearing with aging and is one of the most common conditions affecting older adults [1,2]. In the United States, approximately one in three people between the ages of 65–74 years and one in two adults above 75 years are affected by ARHL [3]. ARHL is typically characterized by deficits in peripheral and central hearing ability [4,5]. Peripheral hearing loss leads to deficits in the detection of sounds (e.g., rustling of leaves, doorbells, safety warnings such as smoke alarms) [6,7] and is often associated with central hearing deficits such as difficulties in discrimination of frequency and timing properties of sound, integration of sounds across both ears, and understanding speech particularly in noisy environments such as restaurants and crowded meeting rooms [8,9,10,11,12]. In fact, difficulty with recognition of speech-in-noise (SiN) is one of the hallmark symptoms of ARHL [8,13]. Numerous studies suggest that even individuals with milder degrees of hearing loss face significant speech recognition difficulties in noisy environments [14,15,16,17]. These deficits persist even when state-of-the-art amplification devices with noise reduction systems are used [18,19], indicating that non-auditory factors may also be contributing to these deficits.

There is growing consensus that alterations in various cognitive faculties, such as episodic and semantic memory, speed of processing, and cognitive control [6,16,20,21,22,23,24,25] may contribute to the challenges experienced by older adults with ARHL [26,27,28,29]. Cognitive control, also referred to as executive control, refers to a broad class of mental operations that allow information prioritization to accomplish current goals [30,31]. Cognitive control includes processes such as *cognitive flexibility*, which is the ability to flexibly shift between tasks or mental sets of information; *inhibition*, which is the ability to suppress irrelevant information to allow processing of relevant information; and *working memory updating*, which is the ability to update and maintain incoming information over a short duration of time [32,33]. These processes are not specific to a particular modality, such as visual or auditory. In the context of ARHL, studies have predominantly investigated alterations in cognitive control processes by examining their associations with measures of peripheral hearing, primarily pure-tone average (PTA) [23,24,28,34,35,36,37,38,39,40], with some recently examining associations with central hearing measures, particularly SiN recognition [35,41,42,43,44,45,46].

Of the cognitive control processes, alterations in working memory updating have been most extensively studied in individuals with ARHL. These studies have typically found alterations on accuracy measures of complex working memory updating tasks, such as Digit Ordering, Visual Letter Monitoring, *n*-back, and Reading Span [34,35,46,47,48]. These deficits have been found in both auditory [34,37] and visual [45,46,47,49] modalities in individuals with varying degrees of hearing loss, including mild [34,35,37,40,45,50,51,52], moderate [34,35,40,45,50], moderately severe [40,45,50], and profound hearing loss [50], with greater severity of peripheral hearing loss associated with worse performance on updating measures. It is important to note that studies have found deficits in complex auditory working memory updating tasks despite presenting stimuli at suprathreshold levels [34,37], indicating that alterations in working memory updating were not solely due to peripheral hearing deficits. A smaller subset of studies has found associations between working memory updating in ARHL and measures of complex SiN recognition, a metric of central hearing ability in ARHL [40,45,46,49,53,54], supporting a link between alterations in working memory updating and central hearing ability.

Relative to working memory updating, fewer studies have examined cognitive flexibility in ARHL, with most using visual tasks. These studies have typically reported poorer accuracy on visual substitution measures such as the Digit Symbol and Letter Digit Substitution tests [23,55,56,57,58], as well as longer completion time on the Trail Making Test-B (TMT-B) [24,38,39,59] in older adults with ARHL compared to normal hearing (NH) controls. Studies have also found significant associations between peripheral hearing loss and performance on measures of cognitive flexibility. In particular, greater severity of peripheral hearing loss has been associated with decreased accuracy on substitution tests and longer completion time on TMT-B. Similar findings have been observed in longitudinal studies involving older adults with ARHL ranging from mild [24,60] to severe degrees of hearing loss [24]. Specifically, older adults with greater hearing loss severity have been found to have poorer accuracy on substitution tests over time despite hearing aid use. A handful of studies have also found a relationship between central hearing and cognitive flexibility in older adults with ARHL. Worse SiN recognition has been linked to decreased accuracy on substitution test [58] and longer completion time on TMT-B [41,42], especially when switching between multiple speech talkers was required [61], suggesting that slowing in cognitive flexibility is also related to central hearing ability in this population.

Also, few studies on ARHL have examined inhibition and the findings are rather inconclusive [24,28,37,38,39,43,44,58,59,62]. While some have reported impairments in performance on visual Stroop measures, such as completion time [39], accuracy [24], and Stroop effect scores (which account for confounds of reading speed) [37] in those with mild [24,37,39] to severe [24] peripheral ARHL as compared to NH controls, others have not found any group differences on measures of Stroop [38,59]. Similarly, associations between peripheral hearing and inhibition are inconsistent. Some studies have found that greater hearing severity was related to longer completion times [39], lower accuracy [24], and worse Stroop effect scores [28,58], whereas others have not reported such significant findings using the visual Stroop [37,38,60] and adapted Simon task [62]. Evidence for associations between central hearing function measured using SiN recognition and inhibition in ARHL is inconclusive. While some have found significant associations between SiN recognition and the Stroop effect [28,43,58] and Simon effect scores [62], others have found no association between SiN recognition and performance on the Stroop task [43,44] in older adults with ARHL. 

Given the discrepancy in findings related to cognitive flexibility and inhibition in individuals with ARHL, the goal of the current study was to concurrently examine these two processes in individuals with unaided mild ARHL compared to age- and education-matched NH controls. Additionally, we examined associations between measures of peripheral and central hearing and performance on tests of cognitive flexibility and inhibition. We hypothesized that (1) individuals with unaided mild ARHL would perform worse on measures of both cognitive flexibility and inhibition as compared to NH controls, (2) poorer peripheral hearing would be associated with poorer performance on measures of cognitive flexibility and inhibition, and poorer central hearing would be associated with worse performance on measures of cognitive flexibility and inhibition. Visual tasks of cognitive flexibility and inhibition were used to allow for comparison of our findings to existing literature.

## 2. Materials and Methods

### 2.1. Participants

Participants included 21 older adults with unaided bilateral mild age-related sensorineural hearing loss (12 female; mean age: 71.29 ± 7.90 years; mean education: 17.76 ± 3.43 years) and 18 age- and education-matched NH controls (12 female; mean age: 67.11 ± 5.46 years; mean education: 17.83 ± 1.65 years). Demographic information for both groups is reported in Table 1. All participants were right-handed, native English speakers, and had normal or corrected vision with no history of learning disabilities, communication disorders, neurological disorders, psychiatric disorders, or traumatic brain injury. Individuals with a history of substance abuse, use of psychoactive medications, known etiologies of hearing loss such as noise-induced, injury-related, or ototoxicity, and those with unilateral and/or bilateral continuous tinnitus, hearing aid use, and major vision problems (untreated cataracts, macular degeneration, glaucoma, retinopathy) were excluded. Additionally, participants completed the Montreal Cognitive Assessment [63], a global cognitive screening measure, which was used to rule out individuals with possible cognitive impairment (cut-off score for exclusion: <26; ARHL mean score: 27.05 ± 2.18; NH mean score: 27.78 ± 1.86) and the Geriatric Depression Scale [64] to rule out those with depressive symptoms (cut-off score for exclusion: >5; ARHL mean score: 0.57 ± 0.97; NH mean score: 0.27 ± 0.57). All participants signed a written informed consent in accordance with protocol 17067 approved by the Institutional Review Board of the University of Illinois Urbana-Champaign before completing the study. 

### 2.2. Audiological Evaluation

All participants underwent a comprehensive audiological assessment conducted by two audiologists who are also trained researchers. Assessments included otoscopic evaluation to rule out the presence of outer ear diseases, tympanometry to ensure normal middle ear function, reflexometry to rule out reflex pathway abnormalities, and pure-tone and speech audiometry. Pure tone audiometry was conducted in a sound-treated booth. Air conduction thresholds in each ear were obtained from 0.25 to 8 kHz with insert earphones as transducers using Equinox 2.0 audiometer calibrated (Interacoustics, Eden Prairie, MN, USA) to the American National Standards Institute S3.6 2010 standards [65]. Bone conduction thresholds were obtained from 0.25 to 4 kHz with a bone conduction transducer. All thresholds were determined using the modified Hughson-Westlake method [66] and were measured in decibels hearing level (dB HL). A speech-frequency PTA was calculated for air conduction thresholds at 0.5, 1, 2, and 4 kHz in each ear. Based on extant literature, NH was defined as ≤25 dB HL PTA in the better ear and ARHL was defined as >25 dB HL PTA in the better ear [24,56]. PTA score in the better ear was used as a measure of peripheral hearing.

Speech audiometry was conducted using insert ear transducers (ER-3A, Etymotic Research, Elk Grove Village, IL, USA). Speech Reception Thresholds (SRTs) using spondee words and Word Recognition Score (WRS) using the Northwestern University list 6 [67] were obtained in each ear. Participants’ ability to recognize SiN using the Quick Speech-in-Noise test (QuickSIN) [68] was also assessed. The QuickSIN test was administered in right, left, and both (binaural) ears. The QuickSIN task required participants to repeat back sentences presented against multi-talker babble at signal-to-noise ratios that varied in 5 dB steps from +25 dB to 0 dB. QuickSIN scores were recorded, with higher scores suggesting worse SiN recognition. Binaural QuickSIN score was used as a measure of central hearing. Binaural scores were chosen as they represent the functional performance of using both ears in daily complex listening tasks. 

### 2.3. Cognitive Flexibility and Inhibition Evaluation

All participants completed a battery of cognitive control tests. Cognitive flexibility was assessed using the Verbal Fluency test, including Category Fluency [69] and Letter Fluency [70], the TMT-B [71], and the Delis-Kaplan Executive Function System Stroop task (D-KEFS) [72]. Inhibition was assessed using the D-KEFS Stroop task and two experimental Go/NoGo tasks [73,74,75,76]. Visual measures of cognitive flexibility and inhibition were selected in order to compare our findings with current studies that have largely used visual measures of cognitive control, as well as to minimize any confounds related to unaided hearing loss. 

#### 2.3.1. Verbal Fluency

As part of Verbal Fluency, both Category and Letter Fluency tasks were administered. Category Fluency required participants to recall as many animals as possible in one minute and Letter Fluency involved recall of as many words as possible that began with the letters F, A, and S, with one minute for each letter. The total number of animals recalled and the sum of recalled words beginning with F, A, and S were computed, with higher scores indicating better cognitive flexibility performance. 

#### 2.3.2. TMT-B

TMT-B required participants to draw a line to connect letters and numbers in alternating and ascending order (e.g., 1-A-2-B). Completion times were recorded, with longer completion times indicating poorer cognitive flexibility performance. 

#### 2.3.3. D-KEFS Stroop

Stroop was administered and scores on two conditions, the color-word interference condition and the color-word interference/switching condition, were obtained. The interference condition required participants to say out loud the ink color in which the color name was written out (e.g., say *red* for color name blue written in red ink) as quickly and accurately as possible. In the interference/switching condition, participants were required to switch between saying the ink color (e.g., say *red* for color name blue written in red ink) and reading the color name itself (e.g., say *blue* for color name blue written in red ink). Switching was cued by boxes drawn around certain words (boxed words require reading the color name) and not around others (unboxed words require saying the ink color). Completion times were recorded for both interference and interference/switching conditions. Mixing cost was calculated by subtracting the completion time on the interference condition from the interference/switching condition. Completion time on the color-word interference condition was used as a measure of inhibition with longer completion times indicating poorer inhibition performance. Mixing cost was used as a measure of cognitive flexibility, with higher Stroop mixing cost reflecting poorer cognitive flexibility.

#### 2.3.4. Go/NoGo Tasks

Participants performed two Go/NoGo tasks, one requiring basic categorization, “Single-Car”, and one requiring superordinate categorization, “Object-Animal”, with the latter being the more complex task requiring careful evaluation of both perceptual and semantic features of stimuli. Details on the development of these tasks can be found in Maguire et al. [73] and both tasks have been successfully used in several studies involving older adults [74,75,76]. In the Single-Car task, participants viewed line-drawn exemplars of a single “car” and a single “dog” 160 and 40 times, respectively, on a computer screen. Basic levels of “car” and “dog” were used for correct discrimination using basic classification (car vs. dog) instead of superordinate classification (vehicle vs. animal). Participants were required to make Go/NoGo decisions, where a button push response was required for “car” (Go) but not for “dog” (NoGo). These decisions were to be as quick and accurate as possible. In the Object-Animal task, participants viewed multiple line-drawn exemplars of “objects” and “animals” 160 and 40 times, respectively. Exemplars of objects consisted of 40 food items, 40 cars, 20 clothing items, 20 kitchen items, 20 human body parts, and 20 tools, and exemplars of animals consisted of items with varying visual typicality, such as cat, snake, elephant, and lobster. Participants were asked to make quick and accurate Go/NoGo decisions, where a button push was required for “objects” (Go) but not for “animals” (NoGo). Both the Single-Car and Object-Animal tasks consisted of 80% (160) Go trials and 20% (40) NoGo trials. This distribution was used to accentuate the tendency for pre-potent responses. Order and practice effects were minimized by pseudo-randomization of the sequence of stimuli in each task, and task order was counterbalanced for each participant. For each task, a Compumedics NeuroScan Stim System Switch response pad (button-box) was used to register Go responses and record reaction times (RTs), and error rate was calculated for Go and NoGo responses.

### 2.4. Statistical Analysis

The data were analyzed using IBM SPSS Statistics (Version 26, IBM Corp., Armonk, NY, USA). General linear models (GLMs) were used to examine between group (ARHL/NH) differences in demographic and audiological factors. GLMs were also used to examine group differences in performance on measures of cognitive control tasks with group (ARHL/NH defined on basis of PTA) as a between-subject variable and measures of cognitive flexibility (total number of words recalled on Verbal Fluency tasks; completion time on the TMT-B; Stroop mixing cost) and inhibition (completion time on the color-word interference condition of the Stroop task; RTs/error rate on trials of Go/NoGo tasks) as within-subject variables. Bonferroni corrections were used to correct for multiple comparisons with alpha set at 0.05. The *p*-values reported in the Results section are significant effects derived from *F-* statistics of contrasts of experimental factor means.

We conducted primary and secondary analyses to examine correlations between hearing and cognitive control measures. For primary analyses, Pearson’s method was used to examine zero-order correlations between peripheral hearing ability (assessed using PTA) and performance on cognitive flexibility and inhibition measures. With regard to Go/NoGo tasks, correlational analyses were restricted to NoGo trials since they reflect inhibition as opposed to Go trials which reflect response execution [77]. Given that central hearing is closely associated with peripheral hearing [27,78], we conducted partial correlations to examine the relationship between central hearing ability and performance on cognitive flexibility and inhibition measures while controlling for peripheral hearing ability. 

As part of our secondary analyses, we examined associations between low- and high-frequency peripheral hearing loss and performance on measures of cognitive flexibility and inhibition. These secondary analyses were motivated by an emerging body of research suggesting that these sub-types of hearing loss are distinctly related to cognition [79,80,81]. We derived low- and high-frequency hearing measures from pure tone thresholds using the method outlined in Eckert et al. [80,81] (see Supplementary Materials for details). This approach involves calculation of low- and high-frequency hearing based on a factor analysis of pure tone thresholds from 852 older adults (mean age = 69.92 ± 7.24 years) who participated in a study on ARHL [82,83]. However, unlike the approach used in Eckert et al. [80,81], wherein low- and high-frequency hearing across the right and left ear were averaged, we calculated low- and high-frequency hearing for the ear with better PTA. We then examined zero-order correlations between low-frequency hearing and performance on cognitive flexibility and inhibition; and high-frequency hearing and performance on cognitive flexibility and inhibition measures. 

For all correlational analyses, Bonferroni corrections were used to correct for multiple comparisons with alpha set at 0.05. All correlational analyses were conducted after dropping outliers (±2 standard deviations from the mean score of the variables being assessed). Accordingly, we dropped two outliers each for PTA, Binaural QuickSIN, Category Fluency, Stroop mixing cost, error rate on NoGo trials of the Object-Animal Task as well as low- and high-frequency hearing measures; and one outlier each for TMT-B, Letter Fluency, interference condition of D-KEFS Stroop task, and error rate on NoGo trials of the Single-Car task. 

## 3. Results

### 3.1. Group Differences

#### 3.1.1. Audiological Measures

**Figure 1:**
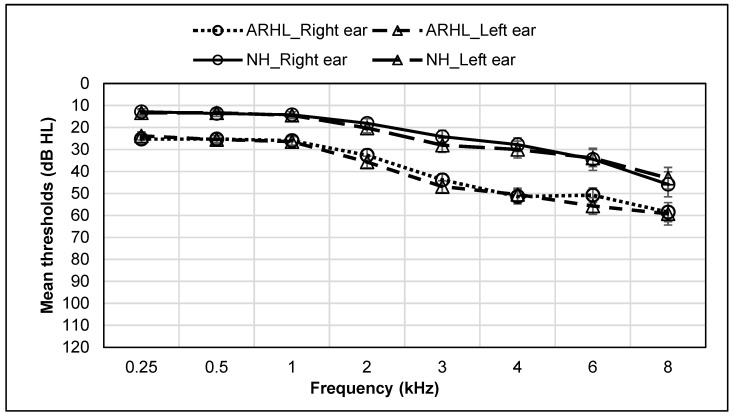
Audiogram for ARHL and NH groups. Average of air conduction hearing thresholds for both ears across frequencies in ARHL and NH groups. Error bars represent standard errors.

Main effects of group were observed for the following audiological measures: PTA, *F*_(1,37)_ = 62.49, *p* < 0.001; Right ear SRT, *F*_(1,37)_ = 19.49, *p* < 0.001; Left ear SRT, *F*_(1,37)_ = 36.66, *p* < 0.001; Right ear QuickSIN score, *F*_(1,37)_ = 14.40, *p* = 0.001; Left ear QuickSIN score, *F*_(1,37)_ = 8.96, *p* = 0.005, and Binaural QuickSIN score, *F*_(1,37)_ = 8.63, *p* = 0.006. Significantly higher PTA, SRT, and QuickSIN scores were observed for the ARHL compared to the NH group. All other effects were not significant. Group means for all audiological measures are reported in Table 2. Audiograms for NH and ARHL groups are shown in Figure 1.

#### 3.1.2. Cognitive Flexibility and Inhibition Measures

A main effect of group was observed for completion time on the Stroop color-word interference condition, *F*_(1,37)_ = 6.14, *p* = 0.018, with significantly longer completion times in the ARHL group compared to the NH group. A main effect of group was observed for error rate on NoGo trials for both Single-Car, *F*_(1,37)_ = 5.10, *p* = 0.030, and Object-Animal tasks, *F*_(1,37)_ = 6.45, *p* = 0.016, with higher error rate in the ARHL group compared to the NH group. All other effects were not significant. Group means for all cognitive control measures are reported in Table 3. 

### 3.2. Correlations

Several significant associations between hearing measures and measures of cognitive flexibility and inhibition were observed. With regard to our primary analyses related to cognitive flexibility, we found a significant positive correlation between PTA and completion time on the TMT-B, *r*(32) = 0.36, *p =* 0.034. When partial correlations between binaural QuickSIN score and measures of cognitive flexibility were examined controlling for PTA, a significant positive correlation between binaural QuickSIN score and TMT-B, *r*(31) = 0.57, *p <* 0.001, and a significant negative correlation between binaural QuickSIN score and Letter Fluency score, *r*(31) = −0.36, *p* = 0.038, were observed. Our secondary analyses found no significant correlations between measures of low- and high-frequency hearing and cognitive flexibility.

**Figure 2:**
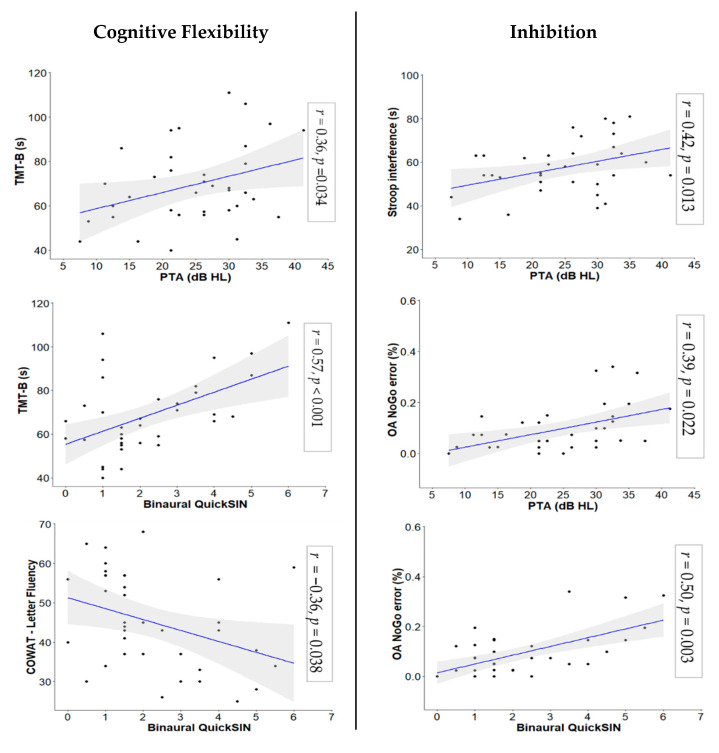
Significant Relationships between Hearing and Cognitive Control Variables. PTA (dB HL) = Pure-Tone Average (decibels hearing level); QuickSIN = Quick Speech-in-Noise [68]; TMT-B = Trail Making Test B [71]; COWAT = Controlled Oral Word Association Test [70]; OA = Object-Animal Task.

On our primary correlational analyses involving inhibition, we found significant positive correlations between PTA and completion time on the Stroop color-word interference condition, *r*(31) = 0.42, *p* = 0.013, and between error rate on NoGo trials of the Object-Animal task, *r*(31) = 0.39, *p =* 0.022. When we examined partial correlations between binaural QuickSIN score and measures of inhibition while controlling for PTA, we observed a significant positive correlation between binaural QuickSIN score and error rate on NoGo trials of the Object-Animal task, *r*(30) = 0.50, *p* = 0.003. Additionally, as part of our secondary analyses, we found a significant positive correlation between better ear high-frequency hearing and completion time on the Stroop color-word interference condition, *r*(31) = 0.46, *p* = 0.006. No other correlations were significant. Scatter plots for all significant associations with primary analyses are shown in Figure 2 (see Supplementary Figure S1 for scatter plots for non-significant associations). All correlations with primary analyses are reported in Table 4 (see Supplementary Table S2 for associations with secondary analyses).

## 4. Discussion

This study examined differences in performance on measures of cognitive flexibility and inhibition between older adults with unaided mild ARHL and NH controls, and associations between measures of peripheral hearing, central hearing, and measures of cognitive flexibility and inhibition. As expected, we found significant differences between ARHL and NH groups on both peripheral (PTA) and central (binaural QuickSIN scores) hearing measures. The ARHL group had higher PTA and binaural QuickSIN scores relative to NH group, reflecting poorer peripheral and central hearing in this population. Three major findings related to the goals of our study emerged: (1) the ARHL group performed worse on measures of inhibition (completion time on the Stroop color-word interference condition and error rate on NoGo trials of Go/NoGo tasks) relative to the NH group, with no significant group differences observed on measures of cognitive flexibility (total number of words recalled on Verbal Fluency tasks, completion time on TMT-B, and Stroop mixing cost), (2) significant associations were observed between measures of hearing and cognitive flexibility (completion time on TMT-B and number of words recalled on Letter Fluency), and (3) significant associations were observed between measures of hearing and inhibition (completion time on the Stroop color-word interference condition and error rate on NoGo trials of the Object-Animal Go/NoGo task). Additionally, on secondary analyses, we observed that high-frequency hearing measure was associated with longer completion time on the Stroop color-word interference condition.

### 4.1. Inhibition

Findings related to group differences revealed that ARHL group demonstrated not only slower, but also worse, inhibitory control. On the Stroop color-word interference condition, the ARHL group took longer to complete the task relative to the NH group, which is consistent with the findings of most prior studies [37,39,59], with two exceptions [38,59]. Our finding suggests that the ARHL group required more time to successfully employ inhibitory control to perform the task with the same accuracy level as the NH group. Furthermore, we found that the ARHL group had higher error rates on NoGo trials on both the simpler Single-Car and the more complex Object-Animal Go/NoGo tasks relative to the control group. The ARHL group’s ability to inhibit their pre-potent responses for the infrequently occurring NoGo trials (20% of the total trials) appears to be worse than that of the NH group leading to higher error rates (i.e., false alarms), indicating inhibitory control deficits. 

Although Go/NoGo tasks have been used extensively in studies with normal cognitive aging and various clinical populations [74,75,76,84], they have rarely been used in the context of ARHL with the exception of Kuchinsky et al. [85]. Kuchinsky and colleagues used RTs of Go trials as a measure of vigilance and found no relation between PTA and performance on Go trials. Similarly, we did not find a significant group difference on RTs and error rates on Go trials, which suggests that older adults with mild ARHL were able to successfully allocate their attentional resources similar to controls to respond to the frequently occurring Go trials (80% of the trials). However, Kuchinsky et al. [85] did not analyze performance on the NoGo trials, therefore our findings related to NoGo trials need to be corroborated in future studies. Additionally, they utilized letters as stimuli for their Go/NoGo task whereas the current study used more complex stimuli of line drawings of animals and objects. How task and/or stimulus complexity may affect inhibition performance in individuals with ARHL requires further examination in studies that compare performance on inhibition tasks involving both simple and complex stimuli. Overall, our inhibition-related findings suggest that cognitive alterations in inhibitory control appear to occur even with milder degrees of hearing loss. One could argue that these alterations in inhibition are typical in older adults, as has been extensively documented in the body of work on normal cognitive aging [32,86,87,88,89,90,91,92]. However, given that we included age-matched NH controls, alterations observed in our ARHL group are above and beyond those typically related to normal aging. 

Our primary correlational analyses revealed that inhibition is related to peripheral and central hearing abilities. Our findings showed a positive association between peripheral hearing measure (PTA) and completion time on the Stroop color-word interference condition, where older adults with higher PTA took longer to complete the color-word interference condition (i.e., they were slower). Our findings are consistent with others who have examined associations between peripheral hearing and inhibitory control measures [24,28,39,58]. We also observed positive associations between PTA and error rates, as well as binaural QuickSIN score and error rates on the NoGo trials of the more complex Object-Animal Go/NoGo task. Older adults with higher PTA and binaural QuickSIN scores made more errors (higher false alarms) on the NoGo trials. Given that we did not observe similar associations with the simpler Single-Car Go/NoGo task, this indicates that the link between hearing and inhibition is modulated by task complexity. The Object-Animal task requires a more involved examination of both perceptual and semantic features compared to the Single-Car task. Additionally, given that the significant association between binaural QuickSIN scores and error rates was observed even when PTA was partialled out, the association between central hearing and inhibitory control appears to go beyond peripheral auditory deficits. These findings support work that ties the critical role of inhibition to SiN recognition in younger and older adults with normal hearing [12,93,94], and in those with ARHL [28,43,44]. 

As part of our secondary analyses, we found a significant positive association between high-frequency hearing measure and completion time on the Stroop color-word interference condition, suggesting that poor high-frequency hearing is associated with slower inhibitory control. Our finding converges with a study by Brännström et al. [79] who also found that poorer high-frequency hearing was associated with poorer inhibitory control on a sustained attention response task. However, it is important to note that Brännström et al. [79] analyzed extended high frequencies (10, 12.5, and 14 kHz) in individuals with NH, unlike our high-frequency hearing measure which included frequencies from 0.25 to 8 kHz. Future investigation in this emerging area is required to better understand how hearing is linked to inhibition in older adults with ARHL. 

### 4.2. Cognitive Flexibility

With regard to cognitive flexibility, we found no significant group differences; however, we observed significant associations between peripheral hearing and cognitive flexibility as well as central hearing and cognitive flexibility. We found a significant positive association between peripheral hearing measure (PTA) and completion time on the TMT-B, which is consistent with previous studies using similar measures [23,38,39,59,60]. This finding suggests that decreased hearing ability is related to slowing in cognitive flexibility. It has been suggested that responding during pure-tone audiometry might involve some amount of cognitive flexibility since it involves deciding whether a sound is present or absent on each trial across frequencies [95]. 

We also found a significant positive association between a central hearing measure, binaural QuickSIN score, and completion time on the TMT-B, indicating that poorer recognition of sentences in background noise is associated with slowing in cognitive flexibility. Furthermore, we found a significant negative association between binaural QuickSIN score and number of recalled words on the Letter Fluency task, which suggests that poorer sentence recognition in background noise is also related to alterations in cognitive flexibility. Our findings align with previous studies that have observed associations between worse SiN recognition and longer TMT-B completion times [41,42,61,96] and a lower number of recalled words on the Letter Fluency task [97]. One could argue that the association between binaural QuickSIN score and TMT-B is confounded by speed of processing. A posteriori analysis showed no change in the association between binaural QuickSIN score and TMT-B when speed of processing was controlled for (TMT-A was used as a control variable; TMT-A requires participants to connect only numbers in ascending order, as opposed to shifting between numbers and letters in TMT-B, and indexes processing speed; see Supplementary Table S3). Furthermore, given that the significant associations between binaural QuickSIN scores and completion time on TMT-B and Letter fluency scores were observed even when the effect of PTA was controlled, these findings suggest that the association between central hearing and cognitive flexibility is above and beyond what is linked to peripheral auditory deficits. However, the mechanism and underlying nature of the association between SiN recognition and cognitive flexibility is far from clear and requires further investigation.

Decades of research have suggested that sensory ability, including hearing, is linked to cognitive performance [29,98,99,100,101,102,103,104,105,106] and our study adds to this vast literature. Several theoretical frameworks have long proposed hypotheses to explain the connection between hearing and cognition, such as the Information Degradation, Sensory Deprivation hypotheses [102,105], and the Common Cause hypotheses [102,104,105], but our study was not designed to examine or explore any of these hypothesized connections. Our study was purely exploratory in nature and was motivated by a desire to understand whether we might observe group differences in cognitive flexibility and inhibition between unaided mild ARHL and NH groups in a cross-sectional sample and to understand associations between hearing and cognitive control within this sample. Although we did observe significant findings both in analyses of group differences and correlations, our findings are not generalizable given our small sample size. Replication studies and future investigations with more participants are necessary to truly understand the nature of alterations in cognitive flexibility and inhibitory control in older adults with mild ARHL.

One of the goals of our study was to separately explore how peripheral hearing and central hearing measures varied in their relationship to cognitive flexibility and inhibition. As the first step in this direction, we examined peripheral hearing using PTA, a threshold measure, and central hearing ability using QuickSIN task, a suprathreshold measure. Future work should consider a more comprehensive evaluation of peripheral and central auditory functioning. For example, the QuickSIN task used in this study measured one of the primary central auditory functions, i.e., auditory performance with competing acoustic signals. Examining other central auditory functions such as auditory discrimination and pattern recognition, and temporal and frequency resolution may shed more light on the link between hearing and cognitive control in ARHL and its potential link to cognitive health. This would be especially important in light of increasing evidence that suggests impairment in central auditory functions may be an early indicator of cognitive decline [107,108,109,110,111,112]. 

Our sampling of measures for cognitive flexibility and inhibition was narrow. Concurrent examination of different cognitive control processes such as cognitive flexibility, inhibition, and working memory updating, with multiple measures for each and assessed using both visual and auditory modalities, will be necessary to comprehensively characterize cognitive control alterations in this population. Finally, participation in our study was restricted to those with unaided hearing loss. The impact of aided hearing on cognitive flexibility and inhibition and whether aided hearing helps mitigate these alterations needs further exploration. To fully understand the nature of cognitive flexibility and inhibition alterations in ARHL, a multi-disciplinary investigative approach, including a combination of behavioral, neuroimaging, and electrophysiological techniques in both cross-sectional and longitudinal studies, is critical.

## Figures and Tables

**Table 1:** Demographic Characteristics of Participants.

	ARHL Group	NH Group
Total N	21	18
Age (years)	71.29 (7.90)	67.11 (5.46)
Education (years)	17.76 (3.43)	17.83 (1.65)
Sex	12F/9M	12F/6M

Cells represent mean (standard deviation). ARHL = Age-Related Hearing Loss; NH = Normal Hearing.

**Table 2:** Group Means for Audiological Measures.

	ARHL Group	NH Group	*p*-Value
Better ear PTA (dB HL)	32.61 (6.69)	16.59 (5.82)	0.000 *
Right SRT (dB HL)	31.19 (9.60)	19.72 (5.80)	0.000 *
Left SRT (dB HL)	31.67 (6.58)	19.44 (5.91)	0.000 *
Right WRS (%)	94.29 (10.62)	98.44 (2.79)	0.116
Left WRS (%)	94.67 (8.61)	98.22 (4.79)	0.128
Right QuickSIN	6.00 (3.15)	2.80 (1.79)	0.001 *
Left QuickSIN	5.69 (3.26)	3.02 (2.02)	0.005 *
Binaural QuickSIN	3.69 (2.86)	1.58 (1.08)	0.006 *

Cells represent means of raw test scores (standard deviation). ARHL = Age-Related Hearing Loss; NH = Normal Hearing; PTA (dB HL) = Pure-Tone Average (decibels hearing level); SRT (dB HL) = Speech Reception Threshold (decibels hearing level); WRS (dB HL) = Word Recognition Score (decibels hearing level); QuickSIN = Quick Speech-in-Noise [68]. * p < 0.05.

**Table 3:** Group Means for Cognitive Control Measures.

	ARHL Group	NH Group	*p*-Value
Category Fluency	20.38 (4.96)	22.72 (4.95)	0.150
COWAT-Letter Fluency	43.29 (12.38)	48.28 (12.80)	0.224
TMT-B (s)	77.11 (25.28)	65.27 (16.49)	0.098
Stroop mixing cost	5.90 (17.50)	8.89 (17.54)	0.599
Stroop color-word interference (s)	62.62 (13.41)	53.39 (8.99)	0.018 *
SC RT ^a^ (ms)	350.25 (51.58)	343.51 (35.85)	0.647
SC Go error ^a^ (%)	0.04 (0.10)	0.96 (0.16)	0.306
SC NoGo error ^a^ (%)	0.14 (0.10)	0.08 (0.05)	0.030 *
OA RT ^a^ (ms)	420.85 (40.65)	438.10 (36.74)	0.180
OA Go error ^a^ (%)	0.05 (0.06)	0.04 (0.07)	0.618
OA NoGo error ^a^ (%)	0.18 (0.21)	0.05 (0.05)	0.016 *

Cells represent means of raw test scores (standard deviation). ARHL = Age-Related Hearing Loss; NH = Normal Hearing. ^a^ ARHL Group, *n* = 20. TMT = Trail Making Test [71]; COWAT = Controlled Oral Word Association Test [70]; SC = Single-Car Task; OA = Object-Animal Task; RT = reaction time. * *p* < 0.05.

**Table 4:** Correlations between Hearing and Cognitive Control Measures.

	PTA (dB HL) ^a^	Binaural QuickSIN Score ^b^
Cognitive Flexibility		
Category Fluency	−0.25	−0.26
COWAT-Letter Fluency	−0.21	−0.36 *
TMT-B (s)	0.36 *	0.57 *
Stroop mixing cost	−0.10	0.05
Inhibition		
Stroop color-word interference (s)	0.42 *	0.20
SC NoGo error (%)	0.33	0.41
OA NoGo error (%)	0.39 *	0.50 *

**^a^** Correlations with PTA (dB HL) are zero-order correlation coefficients. **^b^** Correlations with Binaural QuickSIN scores are partial correlation coefficients. PTA (dB HL) = Pure-Tone Average (decibels hearing level); QuickSIN = Quick Speech-in-Noise [68]; TMT = Trail Making Test [71]; COWAT = Controlled Oral Word Association Test [70]; SC = Single-Car Task; OA = Object-Animal Task. * *p* < 0.05.

## Data Availability

The data presented in this study are available on request from the corresponding author. The data are not publicly available due to privacy reasons.

## Supplementary Materials

The following are available online at https://www.mdpi.com/2308-3417/6/1/22/s1, Figure S1: Non-Significant Relationships between Hearing and Cognitive Control Variables, Table S1: Values for Calculation of Low- and High-Frequency Measures, Table S2: Correlations between Low- and High-frequency Hearing Measure and Cognitive Control, Table S3: Correlation after Controlling for Trail Making Test-A.
