# Supplementary material for: Cognitive Flexibility and Inhibition in Individuals with Age-Related Hearing Loss
Source: Geriatrics (Basel). 2021 Mar 5;6(1):22. doi: 10.3390/geriatrics6010022 (PMC8006052; doi:10.3390/geriatrics6010022)
Supplement: Supplementary file 1 [file geriatrics-06-00022-s001.pdf]

Calculation of Low-frequency and High-frequency Measures

We used the methodology detailed in Eckert et al., 2012, 2019 studies for calculating the low- and high-frequency hearing measures for our secondary analyses. The steps to derive these measures are listed as follows: (1) Each participants' pure-tone thresholds for each ear (from 0.25 to 8 kHz frequencies) were standardized to the mean and standard deviation of the corresponding pure-tone threshold from the 852 older adults (columns 2 and 3 in Supplementary Table A, reproduced from Eckert et al., 2012). For example, a participant with a 30-dB HL threshold at 250 Hz would have a standardized score for that threshold equal to 1.047 (x = [30 − 17.37]/ 12.06). (2) This standardized score was then multiplied by the corresponding low- and high-frequency component coefficient from the factor analysis (columns 4 and 5 in Supplementary Table A, reproduced from Eckert et al., 2012). For example, the same participant from above would have a weighted low-frequency score equal to 0.424 (x = 1.047 * 0.405) and a weighted high-frequency score equal to −0.145 (x = 1.047 * −0.139) for the threshold at 250 Hz. (3) We then summed these weighted values across frequencies (0.25 to 8 kHz) for each component, and thus, created the low- and high-frequency hearing measures. (4) Based on the ear which had better PTA, we used better ear low-frequency and high-frequency measures. For example, if a participant had better PTA in the right ear, then we used the low- and high-frequency measures of the right ear.

Supplementary Table S1. Values for Calculation of Low- and High-Frequency Measures.

Descriptive statistics and factor analysis coefficients that can be used to estimate low and high frequency hearing threshold components

			Component score coefficient matrix	
Frequency (Hz)	*Mean*	*SD*	*Low frequency component*	*High frequency component*
250	17.37	12.06	0.405	−0.139
500	17.70	13.29	0.420	−0.137
1,000	20.05	15.48	0.316	−0.039
2,000	29.44	19.97	0.096	0.137
3,000	38.63	22.81	−0.065	0.253
4,000	47.14	23.98	−0.112	0.280
6,000	55.31	24.19	−0.101	0.273
8,000	58.57	23.69	−0.081	0.248

From Eckert et al., 2012. (Table 1, pg. 707). Reprinted with Permission from the Springer Nature Customer Service Center GmbH: Springer Nature. Eckert, M. A., Cute, S. L., Vaden, K. I., Kuchinsky, S. E., & Dubno, J. R. (2012). Auditory cortex signs of age-related hearing loss. *JARO - Journal of the Association for Research in Otolaryngology*, *13*(5), 703–713. https://doi.org/10.1007/s10162-012-0332-5. Copyright Clearance Center, License Number 5010440403768 February 15, 2021.

Supplementary Table S2. Correlations between Low- and High-frequency Hearing Measure and Cognitive Control.

	Low-frequency hearing measure	High-frequency hearing measure
Cognitive Flexibility		
Category Fluency	−.28	.05
COWAT - Letter Fluency	−.06	−.11
TMT-B (s)	.16	.30
Stroop mixing cost	.17	−.26
Inhibition		
Stroop color-word interference (s)	.13	.46*
SC NoGo error (%)	.14	.17
OA NoGo error (%)	.24	.28

Cells represent zero-order correlation coefficients. COWAT = Controlled Oral Word Association Test [70]; TMT = Trail Making Test [71]; SC = Single-Car Task; OA = Object-Animal Task. *$p < .05$

Supplementary Table S3. Correlation after Controlling for Trail Making Test-A.

	Binaural QuickSIN score
TMT-B	.57*

Cell represents partial correlation coefficient after controlling for completion time on TMT-A. QuickSIN = Quick Speech-in-Noise [68]; TMT = Trail Making Test [71]. *$p < .05$

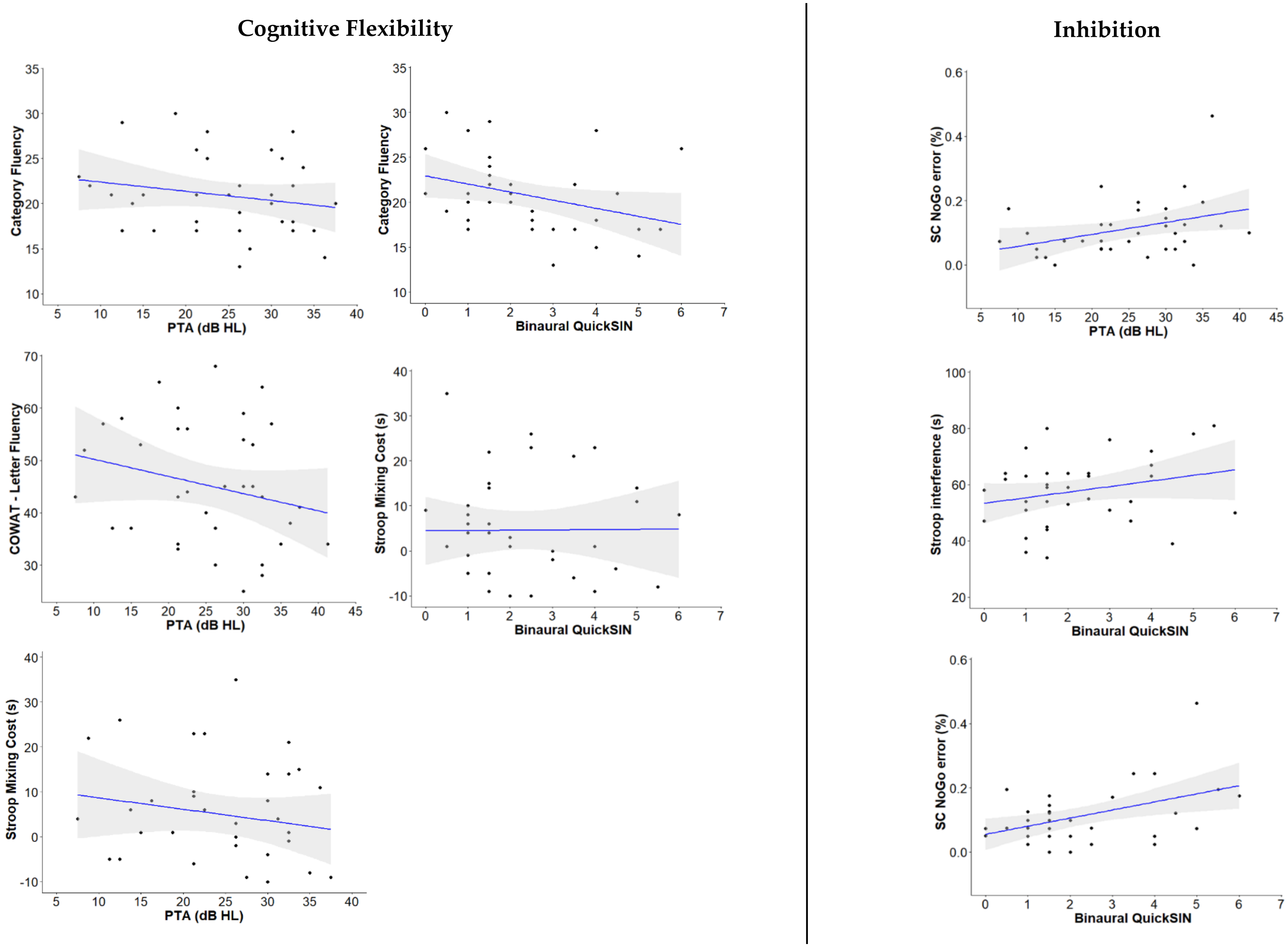

Supplementary Figure S1. Non-Significant Relationships between Hearing and Cognitive Control Variables. PTA (dB HL) = Pure-Tone Average (decibels hearing level); QuickSIN = Quick Speech-in-Noise [68]; COWAT = Controlled Oral Word Association Test [70]; SC = Single-Car Task; OA = Object-Animal Task.
